# Exploring research hotspots and future directions in neural tube defects field by bibliometric and bioinformatics analysis

**DOI:** 10.3389/fnins.2024.1293400

**Published:** 2024-04-08

**Authors:** Rui Cao, Yanbing Su, Jianting Li, Ruifang Ao, Xiangchao Xu, Yuxiang Liang, Zhizhen Liu, Qi Yu, Jun Xie

**Affiliations:** ^1^Department of Biochemistry and Molecular Biology, Shanxi Key Laboratory of Birth Defect and Cell Regeneration, Key Laboratory of Coal Environmental Pathogenicity and Prevention of Ministry of Education, Shanxi Medical University, Taiyuan, China; ^2^Translational Medicine Research Centre, Shanxi Medical University, Taiyuan, China; ^3^Shanxi Bethune Hospital, Shanxi Academy of Medical Sciences, Tongji Shanxi Hospital, Third Hospital of Shanxi Medical University, Taiyuan, China; ^4^Sci-Tech Information and Strategic Research Center of Shanxi Province, Taiyuan, China

**Keywords:** bibliometric, bioinformatics, gene, single nucleotide polymorphism (SNP), neural tube defects (NTDs)

## Abstract

**Background:**

Neural tube defects (NTDs) is the most common birth defect of the central nervous system (CNS) which causes the death of almost 88,000 people every year around the world. Much efforts have been made to investigate the reasons that contribute to NTD and explore new ways to for prevention. We trawl the past decade (2013–2022) published records in order to get a worldwide view about NTDs research field.

**Methods:**

7,437 records about NTDs were retrieved from the Web of Science (WOS) database. Tools such as shell scripts, VOSviewer, SCImago Graphica, CiteSpace and PubTator were used for data analysis and visualization.

**Results:**

Over the past decade, the number of publications has maintained an upward trend, except for 2022. The United States is the country with the highest number of publications and also with the closest collaboration with other countries. Baylor College of Medicine has the closest collaboration with other institutions worldwide and also was the most prolific institution. In the field of NTDs, research focuses on molecular mechanisms such as genes and signaling pathways related to folate metabolism, neurogenic diseases caused by neural tube closure disorders such as myelomeningocele and spina bifida, and prevention and treatment such as folate supplementation and surgical procedures. Most NTDs related genes are related to development, cell projection parts, and molecular binding. These genes are mainly concentrated in cancer, Wnt, MAPK, PI3K-Akt and other signaling pathways. The distribution of NTDs related SNPs on chromosomes 1, 3, 5, 11, 14, and 17 are relatively concentrated, which may be associated with high-risk of NTDs.

**Conclusion:**

Bibliometric analysis of the literature on NTDs field provided the current status, hotspots and future directions to some extant. Further bioinformatics analysis expanded our understanding of NTDs-related genes function and revealed some important SNP clusters and loci. This study provided some guidance for further studies. More extensive cooperation and further research are needed to overcome the ongoing challenge in pathogenesis, prevention and treatment of NTDs.

## 1 Introduction

Neural tube defects (NTDs) are common birth defects, which seriously affects the quality of population. It’s a kind of congenital malformation caused by incomplete closure of neural tube during embryonic development (Copp et al., 2013). NTDs have a very complex sort of etiology including interaction of both genetic and environmental factors, such as intake of high sugar, high calorie or toxic substances during pregnancy which can lead to NTDs (Liu et al., 2018). Although the incidence of NTDs has decreased significantly in recent years, NTDs are still one of the common congenital malformations, and the incidence is just next to congenital heart disease. There are still many scientists focusing on NTDs research.

In terms of prevention, firstly, research on the mechanism of folic acid in preventing NTDs is still ongoing (Dong et al., 2023). However, folic acid cannot prevent all types of NTDs, and finding alternative supplements has become another research hotspot (Greene et al., 2017). Secondly, the discovery of biomarkers and gene mutations related to NTDs is crucial for prenatal diagnosis, and is also one of the research hotspots (Huang et al., 2022). In terms of treatment, such as the Management of Myelomeningocele Study (MOMS) and it’s follow-up (MOMS2), a randomized controlled clinical trial to compare the safety and efficacy of prenatal and standard postnatal repair of myelomeningocele, demonstrated improved outcomes for children who had prenatal repair (Farmer et al., 2018; Rintoul et al., 2020; Houtrow et al., 2021). Besides the groundbreaking work of surgery, cellular therapy has become another emerging treatment method that is being tried and tested in the field of NTDs (Wei et al., 2023).

Currently, bibliometrics has been recognized as the most active discipline branch in the field of information, reflecting the quantitative trend of contemporary disciplines. In the field of life science, bibliometrics has been widely used in the evaluation of disciplines, fields, journals and research institutions (Devos and Menard, 2019; Akmal et al., 2020). For example, in 2022 Mary Nadine Alessandra R. Uy et al. published the frst bibliometric analysis paper on spina bifda in Asia. Based on 652 articles in the Scopus database, they analyzed the publication by different countries, institutions and authors, as well as the cooperation situation, top cited articles, keywords. Providing us the landscape and trajectory of spina bifida research in Asia (Uy and Tantengco, 2022). Our research hopes to provide scientists more information from a global perspective. Relying on bibliometric data mining, a large number of genes, SNPs and other biological data can be obtained. For these data, a series of bioinformatics tools can be used, such as GO and KEGG enrichment analysis for genes, so as to better understand the functions of these genes from a macro perspective. In view of SNP information, SNP density map can be constructed to show the distribution of SNP in different chromosomes and more directly reflect the SNP situation studied in this field. As far as we know, there are relatively few studies on bibliometric analysis combined with bioinformatics analysis in NTDs field. To make more attempts, we aim to explore research hotspots and future directions in NTDs field by the past 10 years bibliometric analysis and bioinformatics analysis with our study.

## 2 Materials and methods

### 2.1 Materials

We chose the Web of Science (WOS) database, TS = (“Neural tube defects “OR “NTDs” OR “NTD” OR “Exencephaly” OR “anencephaly” OR “spina bifida aperta” OR “open spina bifida” OR “Myelomeningocele” OR “Craniorachischisis” OR “Closed spinal lesions” OR “Encephalocele” OR “meningocele” OR “Iniencephaly”), and selected the period from 1/1/2013 to 12/31/2022. Only the records whose document types were “paper,” “review,” “conference paper,” “online publication” and the publication language was “English” were selected. Finally, we downloaded all 7,437 bibliographic records from WOS database in the txt format “full records and references.”

### 2.2 Methods

#### 2.2.1 General situation

General situation included the distribution of publications during the 10 years were analyzed by SCImago Graphica.

#### 2.2.2 Collaboration analysis

**Figure 1:**
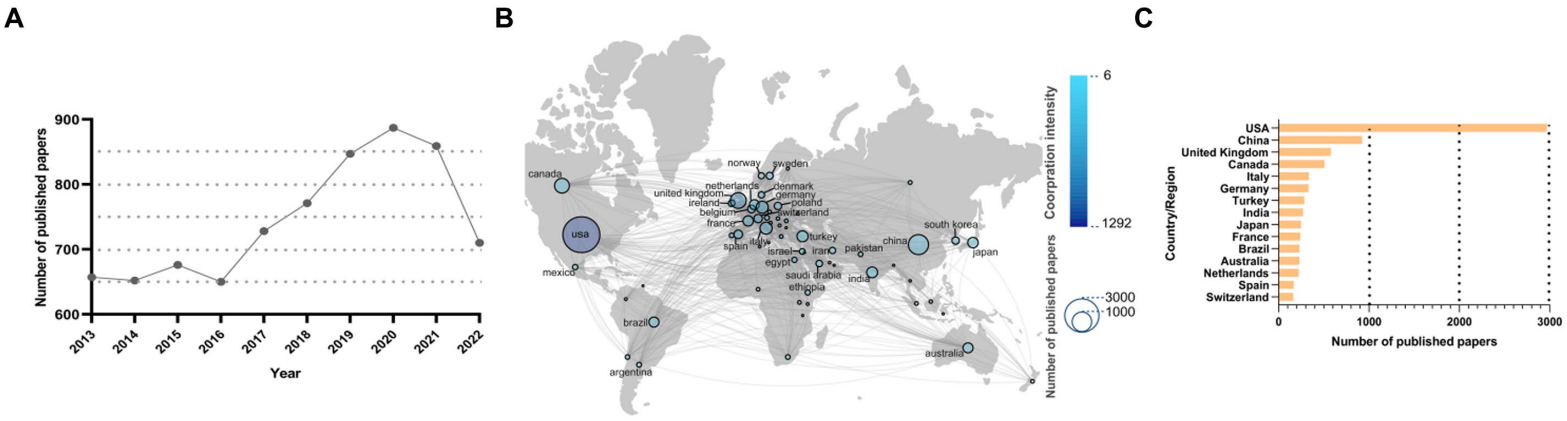
Overall, there has been a growing trend of publication in the field of NTDs in the last decade. Globally, the United States (USA) has the most extensive partnerships with other countries with the highest number of papers published. **(A)** Annual publication volumes in the field of NTDs from 2013–2022. **(B)** The national cooperation network. Different colors represent different cooperation intensity, and the size of the dots represents the number of publications. The names of the top 30 countries with most publications are displayed. **(C)** Top 10 countries with most publications.

**Figure 2:**
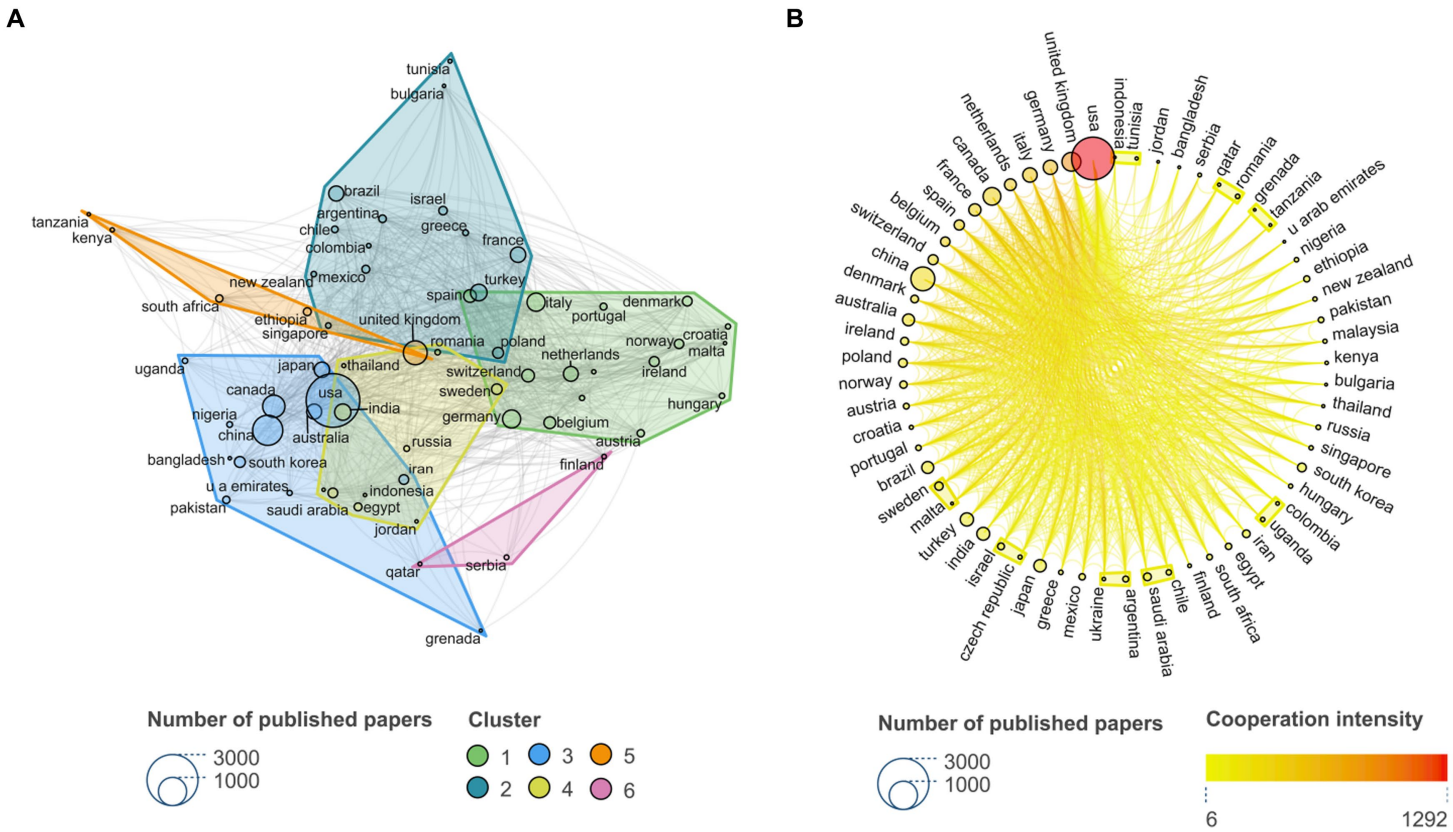
Countries around the world are clustered into six groups to cooperate with each other, with the United States cooperating most closely with other countries. **(A)** Countries collaboration network, within six clusters. Different colors represent different clusters, and the size of the dots represents the number of publications. **(B)** Cooperation intensity map of 61 countries, different colors represent different cooperation intensity, and the size of the dots represents the number of publications.

**Figure 3:**
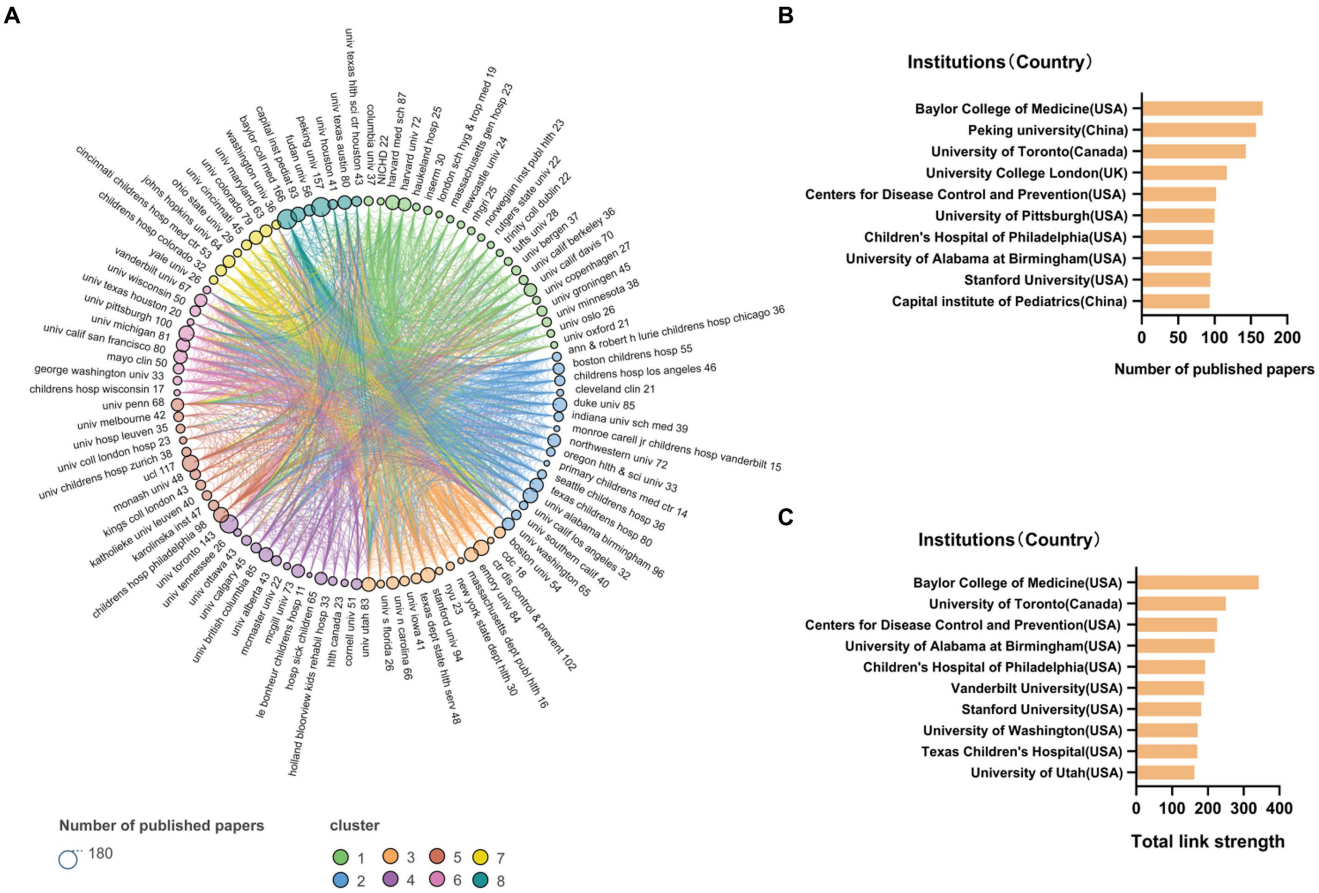
Institutions around the world are clustered into eight groups to cooperate with each other. Baylor college of medicine has the most extensive partnerships with other institutions with the highest number of papers published. **(A)** Institutions collaboration network, within eight clusters. Different colors represent different clusters, and the size of the dots represents the number of publications. **(B)** Top 10 institutions with most publications. Most of them are located in the United States (USA). **(C)** Top 10 institutions with most total link strength. Nine of them are located in the United States (USA).

**Figure 4:**
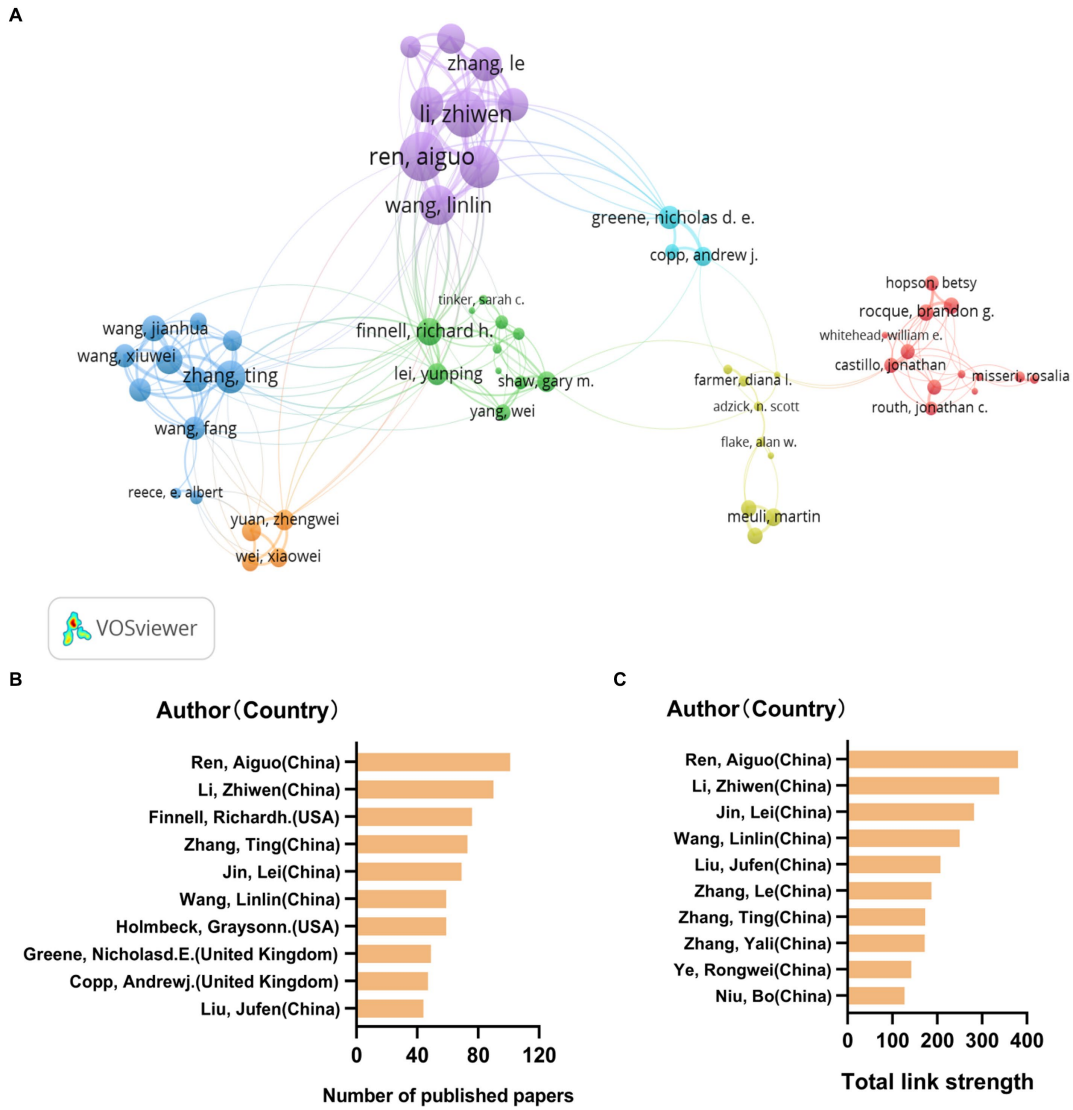
Authors are clustered into seven groups to cooperate with each other. In the top 10 list of publication and collaboration intensity, most authors are from China. **(A)** Author cooperative network. Different colors represent different clusters, and the size of the dots represents the number of publications. **(B)** Top 10 authors with most number of papers published. Most of them are from China. **(C)** Top 10 authors with most total link strength. All of them are from China.

The cooperative network analysis of countries, authors and institutions were analyzed by VOSviewer and visualized by SCImago Graphica (van Eck and Waltman, 2010). Records downloaded from WOS were imported into the VOSviewer software. The setting parameters of were: (1) Collaboration on countries level: maximum number of countries per document is 25; minimum number of documents of a country is 10; 123 countries met the threshold and 61 countries were visualized by VOSviewer’s default setting. The results in gml format generated by vosviewer were saved and imported into SCImago Graphica software for visualization. As shown in Figures 1, 2. (2) Collaboration on institutions level: maximum number of institutions per document is 25; minimum number of documents of an institution is 10; 355 institutions met the threshold and 100 institutions were visualized by VOSviewer. The results in gml format generated by vosviewer were saved and imported into SCImago Graphica software for visualization. As shown in Figure 3A. (3) Collaboration on authors level: the maximum number of authors per paper is 25. The minimum number of document for an author is 20; 64 authors met the threshold and 61 were visualized by VOSviewer’s default setting. As shown in Figure 4A.

#### 2.2.3 Key words analysis

**Figure 5:**
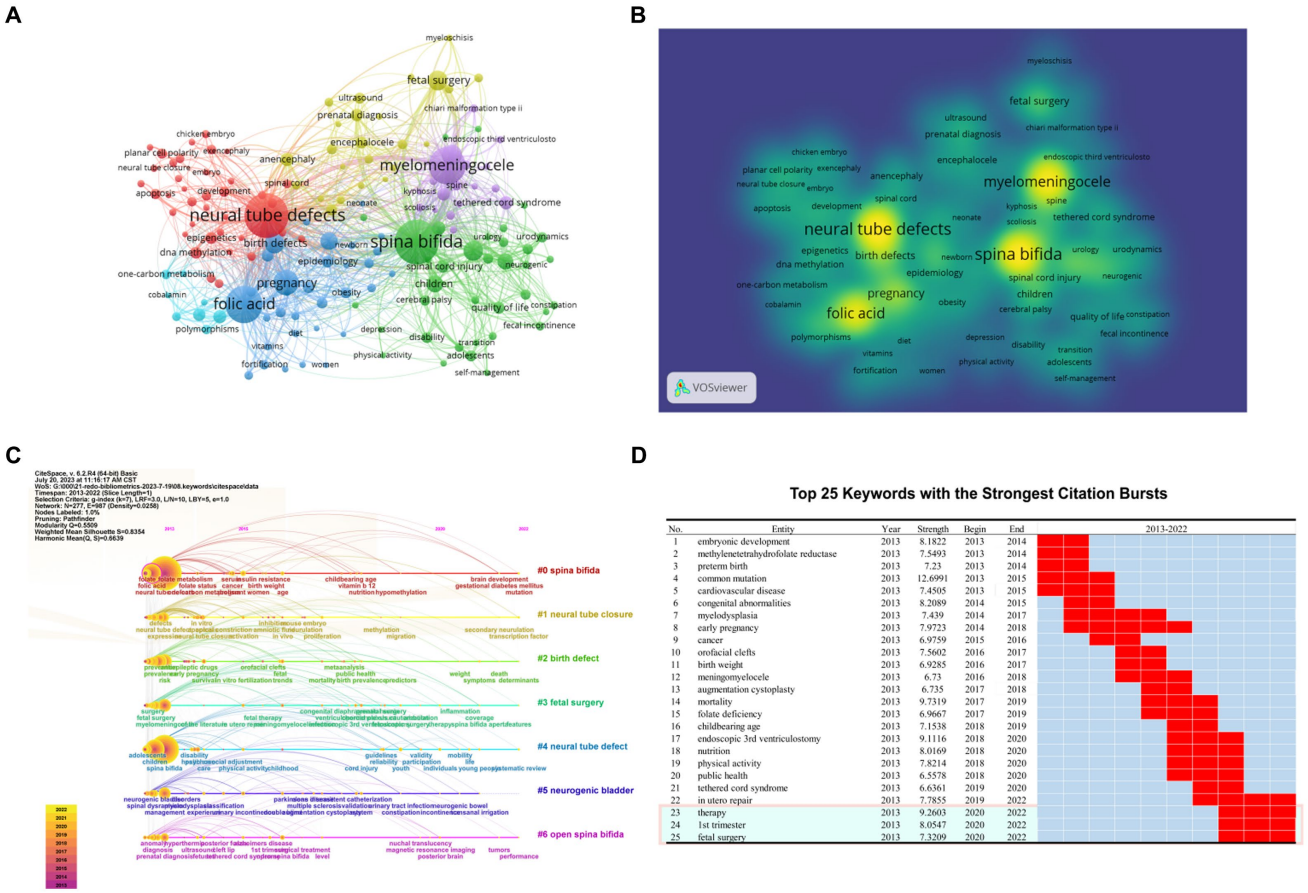
Keyword analysis indicated the research fronts and trend. **(A,B)** Keyword co-occurrence **(A)** and keyword density map **(B)**. They were constructed by Vosviewer. **(C)** Keyword timeline map visualized by Citespace. These results shown in panels **(A–C)** pointed that researches in NTDs field were focus on the genetic and non-genetic causes, the prenatal repair and postnatal treatment for major clinical phenotypes and molecular mechanisms of folic acid prevention. **(D)** Top 25 burst keywords in the field of NTDs. In the past 3 years, keywords like “therapy,” “first trimester,” “*in utero* repair,” “fatal surgery” were with the strongest citation bursts. They indicated the research trend to some extent.

The keywords density, clustering and time line analysis were analyzed by VOSviewer and CiteSpace, The setting parameters of were: (1) VOSviewer: after unifying synonyms, the author keywords were selected for clustering. Minimum number of occurrence of a keyword set to be 20. 157 keywords met the threshold. Both the cluster map and density map were visualized by VOSviewer. As shown in Figures 5A,B. (2) CiteSpace: time slicing is from 2013.1 to 2022.12, and the time cut-off point of analysis is 1 year; g-index *k* = 7. Both the timeline view and burst entries were generated by CiteSpace. As shown in Figures 5C,D.

#### 2.2.4 Biomedical entity analysis

7,437 records were filtered by pubmed ID, 6,781 records with PMID information were gotten to further biomedical entities extract, such as genes and single nucleotide polymorphisms (SNPs) information.

6,781 PMIDs were consolidated into a single txt document, and an automatic batch download program was written using Python language. The PubTator tool was used to extract the biological entity data in available 6,781 literatures, such as gene names and SNP names. All genes and SNPs obtained through the above process were used for further bioinformatics analysis.

#### 2.2.5 Bioinformatics analysis

GO and KEGG enrichment were performed on the obtained gene set using R packages such as enrichplot, clusterProfiler, GOplot, topGO, circle, and ComplexHeatmap. The position of SNPs on chromosomes was obtained using VEP.^1^ CMplot R package and MG2C^2^ visualize the distribution results.

## 3 Results

### 3.1 Wave-like rising trend of global publications with the United States leading the world in the past decade

From 2013 to 2022, the number of published papers in this field showed a “wavy” rising pattern, and there was a significant increase after 2017. However, the number of published papers in 2022 decreased. There are several possible reasons for the decline: (1) Due to the complexity of the disease, there have been no breakthroughs in the etiology, early diagnosis, and treatment of NTDs. (2) Some articles submitted in 2022 have not been published yet. (3) The global birth rate and the incidence of NTDs have continued to decline in recent years, which may have resulted in the scientific community’s attention to neonatal diseases being less than that paid to other diseases, such as geriatrics and cancers, and consequently the number of articles published in the field has also declined. As shown in Figure 1A. Although the incidence of NTDs has been decreasing in recent years (Lu, 2023), the increasing number of publications reflects the continuous scientific research in the field of NTDs. Globally, the United States (USA) ranks first with 2,969 publications. The remaining top 5 countries include China (2,285 publications), the United Kingdom (1,424), Germany (1,129), Japan (975). USA also has the closest cooperation with other countries. As shown in Figures 1B,C.

### 3.2 Highly cited papers in NTDs field (2013–2022)

In general, highly cited papers reflect the attention scientist groups divert to hotspots and urgent academic topics. In our study, 34 papers were provided by WOS system shown as in Supplementary Table 1.

#### 3.2.1 Systematic review

Copp AJ 2013, 2014, and 2015 successively published reviews on NTDs and spina bifida, systematically describing the research status and unsolved problems in this field (Copp et al., 2013; Greene and Copp, 2014; Copp et al., 2015). Zaganjor, I, Dewan, MC et al. carried out a global epidemiological study on NTDs and hydrocephalus, and gave a systematic evaluation on the morbidity, mortality, and regional distribution (Zaganjor et al., 2016; Dewan et al., 2018). In addition, meta-analyses of certain countries/regions have also been cited frequently, such as USA and Botswana (Honein et al., 2017; Reynolds et al., 2017; Zash et al., 2019).

#### 3.2.2 Research on pathogenic factors of NTDs

Gernand AD et al. proposed that the intake of trace elements (Bailey et al., 2015; Gernand et al., 2016; Mousa et al., 2019), nitrate in drinking water (Ward et al., 2018), vegetables and fruits (Saini et al., 2015), and dietary choline (Wiedeman et al., 2018) during pregnancy were all related to NTDs. Mycotoxins have been indicated to disrupt sphingolipid metabolism, thereby disrupting folate transport across cell membranes (Marin et al., 2013; Marroquín-Cardona et al., 2014; Wu et al., 2014; Kamle et al., 2019). Maternal obesity and aluminum exposure are also associated with high-risk of NTDs (Willhite et al., 2014; Marchi et al., 2015).

In addition to nutritional and environmental factors, many studies on pathogenicity factors have been carried out from the perspectives of genetics and molecular biology. As Hamann, J pointed out during avian neural tube closure, ADGRC1 is involved in the contraction of planar polarized actin-myosin cables and apical constriction of neuroepithelial cells which facilitating the vital inward bending of the neural plate. Mutant in ADGRC in mice and mutations in ADGRC1 in human also cause severe developmental defects such as the neural tube defects (Hamann et al., 2015). Genes and metabolism related to folic acid have also been widely studied and cited, such as one-carbon metabolism, MTHR. Their dysfunction are related to the pathogenesis of neural tube defects (Gatt et al., 2015). Many signaling pathways, such as the Wnt signaling pathway, also mediate the formation of neural tube (Zhou et al., 2014).

#### 3.2.3 Prevention of NTDs

Although folic acid cannot prevent all types of NTDs, supplementing folic acid can significantly reduce the birth rate of NTDs, which has been recognized worldwide. Kancherla V consider that Mandatory food fortification with folic acid is a safe, cost-effective, and sustainable intervention to prevent NTDs and urgently call on the World Health Assembly to adopt a resolution on universal mandatory fortification of folic acid (Kancherla et al., 2022). The levels of maternal folic acid and homocysteine have become biomarkers for the risk of fetal NTDs (Bailey et al., 2015; Smith and Refsum, 2021).

### 3.3 Extensive collaboration on a global scale, institutions from the United States and scientists from China cooperating more closely with other partners

The countries/regions, institutions, and authors included in the literature are visually analyzed by vosviewer and SCImago Graphica. Each dot in the map represents a country/region or institution or author. The connection between dots represents the connection or cooperative relationship between countries/regions/institutions/authors. From 2013 to 2022, a total of 123 countries/regions, 6,750 institutions, and 30,665 authors participated in the publication of NTDs theme papers.

123 countries were grouped into 6 clusters. As shown in Figure 2A. Total link strength represented the frequency of collaboration between the country and other countries. In cluster 1, the top5 included Germany, Italy, Netherlands, Spain, Belgium. In cluster 2, the top5 included France, Poland, Brazil, Turkey, Israel. In cluster 3, the top5 included USA, Canada, China, Australia, Japan. In cluster 4, the top 5 included Sweden, India, Saudi arabia, Egypt, Russia. In cluster 5, the top 5 included the United Kingdom, South Africa, Kenya, Ethiopia, Tanzania. According to the intensity of cooperation, the top 10 are the United States, the United Kingdom, Germany, Italy, Canada, Switzerland, France, the Netherlands, Spain, and China. As shown in Figure 2B. From the country composition of each cluster, it could be seen that cooperation in the field of NTDs was not limited to certain countries or certain regions. Global scientists have breaked geographical limitations and cooperated closely with other researchers all over the world, regardless of the differences of national development levels, NTDs incidence rates, and geographical distance. From the perspective of the frequency of collaboration, countries with close cooperation with other countries were not limited to developed countries such as the American and European countries, and developing countries in Asia and Africa also had close cooperation networks with the rest of the world.

Among the 6,750 institutions, 355 met the default parameter values and were aggregated into 8 clusters, and 100 were selected for display. As shown in Figure 3A. Among top10 institutions with the most publications, 6 are from the USA, while the remaining four are Peking University and Capital Institute of Pediatrics from China, University of Toronto from Canada, University College London from UK, as shown in Figure 3B. According to the total_link_strength, 9 of the top 10 institutions are from the USA. As shown in Figure 3C. Six institutions in the USA are ranked in the top 10 in terms of both the number of publications and the link strength, namely: Baylor College of Medicine, Centers for Disease Control and Prevention, the University of Alabama at Birmingham, children’s hospital of philadelphia, Stanford University, University of Toronto.

The cooperation of 30,665 authors were analyzed. The maximum number of authors per paper was set to be 25. The minimum number of document for an author was set to be 20.64 met the threshold. It is clustered into 7 clusters shown as in Figure 4A. The core researchers of each cluster are: Rocque, Brandong, Finnell, Richardh, Zhang, Ting, Meuli, Martin, Ren, Aiguo, Greene, Nicholasd, Yuan, Zhengwei. Among them, Zhang Ting, Ren Aiguo, Finnell Richardh. And Greene Nicholasd ranked in the top 10 of the number of published papers in the past 10 years as shown in Figure 4B. It is worth noting that according to the total link strength ranking, the top 10 authors are all from China, including the Peking University team led by Ren Aiguo and the Capital Institute of Pediatrics team led by Zhang Ting as shown in Figure 4C.

### 3.4 The continuing hotspots of understanding, preventing and proper treatment in NTDs field

Using CiteSpace and VOSviewer to analyze keywords co-occurrence, density, clustering and emergence can help understand the research hotspots, Frontiers, and trends in this field. VOSviewer statistics show that there are 12,724 keywords in 7,437 articles, of which 95 keywords appear 30 times or more, 157 keywords appear 20 times or more, and 361 keywords appear 10 times or more. The 157 keywords that appeared more than 20 times were clustered into 6 clusters as shown in Figure 5A, different colors represent different clusters. Cluster 1 mainly involves the general situation of NTDs field and epigenetic, methylation, embryonic development, etc. Cluster 2 mainly consists of neurogenic diseases caused by NTDs such as spina bifida and other neurogenetic diseases. Cluster 3 mainly focuses on folic acid prevention and pregnancy management. Cluster 4 mainly involves surgical treatment of non fatal malformations. Cluster 5 mainly focuses on the study of meningomyelocele syndrome. Cluster 6 mainly focuses on one of the important mechanisms involved in NTDs: one-carbon Metabolism. Keyword density distribution in Figure 5B shown “neural tube defects,” “spina bifida,” “myelomeningocele,” “folic acid” with the largest nodes and the highest density.

Keyword-based logarithmic likelihood ratio (LLR) is used to cluster keywords and draw a timeline keyword map to show the differences in time and research progress of each cluster. The clustering map is evaluated by the clustering modular value Q (Modularity Q) and the clustering contour index S (Mean Sihouette). The larger the S value is the higher the similarity of the cluster members is; *Q* > 0.3 represents that the structure of division is significant S > 0.5 represents that clustering is reasonable and S > 0.7 represents that clustering is convincing (Aryadoust et al., 2020).

In our study, keyword clustering Modularity *Q* = 0.5509 > 0.3, Mean Silhouette = 0.8354 > 0.5, show that keyword-clustering structure is clear, clustering is reasonable, and reliability is high. By LLR calculation of keywords, 7 natural clusters are obtained as shown in Figure 5C. Different from the Vosviewer’s results, neural tube closure was clustered separately, suggesting the importance of neural tube development and embryonic development in the field of NTDs. From the timeline, it can be seen that folate metabolism is still an important research direction in this field. Figure 5D shows the top 25 emerging words in this field, which shows the research hotspots in this field over the past 10 years, and can predict the trend in the future to some extent. In the past 3 years, early diagnosis and treatment have continued to be important issues, with keywords including: therapy, first trimester, *in utero* repair, and fatal surgery. Other emerging words are also in line with this research theme and are hot topics in a certain time.

**Figure 6:**
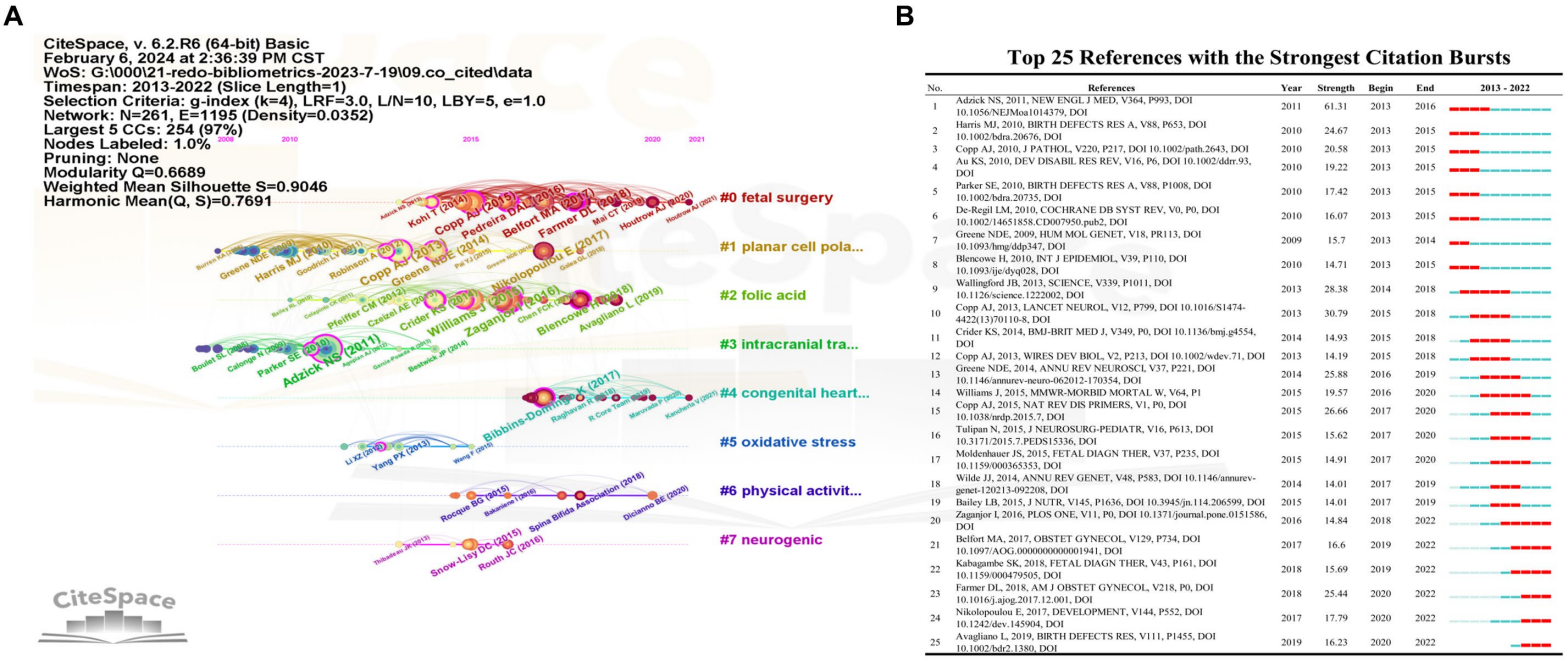
Co-cited references analysis indicated the research fronts and trend. **(A)** The timeline map of co-cited references. **(B)** Top 25 references with the strongest citation bursts. All results shown that the continuing hotspots in NTDs field included the understanding, preventing and proper treatment of NTDs.

In addition to this, we used CiteSpace to do co-cited references should also be analyzed as shown in Figure 6. The timeline map of co-cited references shown in Figure 6A, top 25 references with the strongest citation bursts shown in Figure 6B. As shown in Figures 5, 6, the continuing hotspots in NTDs field included the understanding, preventing and proper treatment of NTDs.

So far, all results suggest that NTDs is a complex polygenic disease caused by the interaction of environmental factors and genetic factors, but the exact molecular mechanism of its occurrence is still unclear. Environmental factors related to the occurrence of NTDs mainly include: nutritional factors of pregnant women during perinatal pregnancy, negative life events, diabetes, obesity, high fever, infection, medication history and radiation, etc. (Finnell et al., 2021). The genetic factors associated with the occurrence of NTDs are very complex, because neural tube formation is an extremely complex developmental biological process involving the precise spatiotemporal expression of a large number of genes (Copp and Greene, 2013; Nikolopoulou et al., 2017; Avagliano et al., 2019). Alterations in one or some of these genes, or in their upstream regulators, can lead to the NTDs (Copp and Greene, 2010). So far, no major gene has been identified. Although folic acid can prevent 30–50% NTDs, the mechanism has not yet been clarified, and a great deal of research has centered on the relationship between disorders of folate metabolism and NTDs. As a 1-carbon source, folic acid plays an important role in the maintenance of methylation, a large number of studies have been carried out on the mechanism of methylation and epigenetic in NTDs field (Cao et al., 2022).

At present, the prenatal diagnosis of NTDs mainly relies on ultrasound, which can not meet the requirements of early diagnosis. Identification of maternal peripheral blood molecular markers has been a promising strategy for non-invasive prenatal testing. In the 1870s, the increase in the alpha-fetoprotein (AFP) concentration in the maternal serum was first to be a indicator for anencephaly (Krantz et al., 2010), and it is still widely used today. It is of great significance to find more earlier and specific diagnostic markers for NTDs. At present, a large number of studies on NTDs’ biomarkers have been carried out based on various methods such as proteomics, multi-omics, and meta-analysis (An et al., 2015; Bailey et al., 2015; Tang et al., 2015; Huang et al., 2022), and the results of these studies have yet to be verified in a wider population. In recent years, it has been found that exosomes may be an important way for fetus-derived molecules to enter maternal peripheral blood, and the screening of molecular markers in exosomes has also become an important way to find malformation specific molecular indicators (Lamont et al., 2020). Recently CORO1A and DNM2 in serum exosomes are found as biomarkers of NTDs (Wang et al., 2022).

Scientists’ efforts in the treatment of NTDs have also continued. The results obtained through the Randomized multicenter Management of Myelomeningocele Study (MOMS Trial) are promising. The MOMS trial has shown that prenatal surgery performed before the 26th week of pregnancy can reduce the risk of neonatal death in the case of meningomyelocele (Moldenhauer and Adzick, 2017). Fetal endoscopic repair is a promising alternative to open fetal MMC repair with a lower risk of uterine rupture (Kabagambe et al., 2018). The potential therapeutic approach of stem cells has also been concerned. In the *ex vivo* rat embryonic NTD model, it was found that BMSCs injected into the amniotic cavity could spontaneously migrate into the defective nerve tissue (Wei et al., 2020). A range of treatments, such as prenatal repair, postnatal surgery, and stem cell therapy are all still being further studies. In short, the etiology, early diagnosis, treatment and other aspects of NTDs have their own research difficulties and directions, and more progress will be made in the future with the advancement and application of various molecular biology tools.

### 3.5 Functional enrichment analysis of NTDs-related genes and important pathways, SNP clusters and loci were found

**Figure 7:**
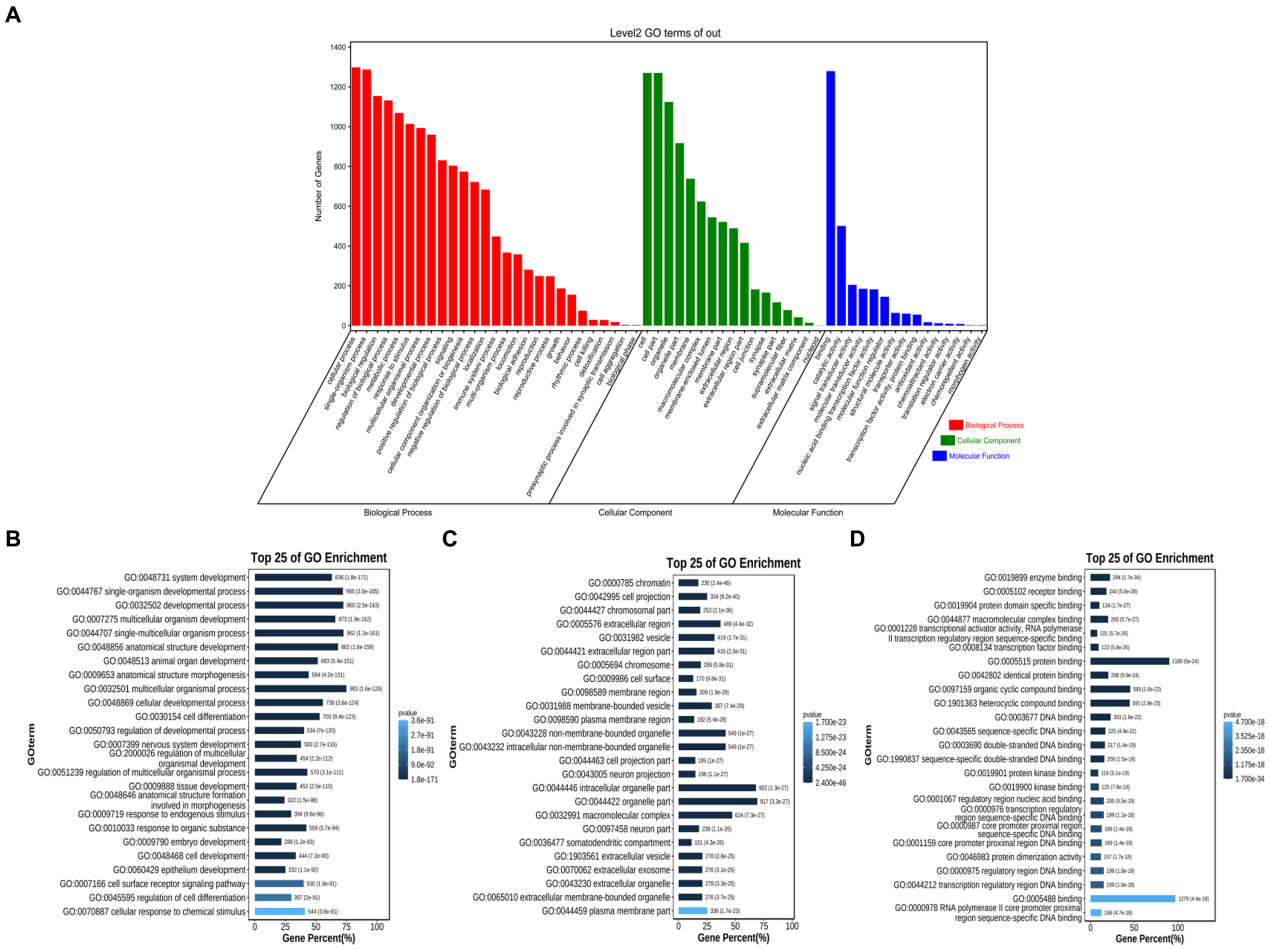
GO enrichment analysis result. **(A)** GO functional classification. Red represents biological process level, green represents cellular component level, and blue represents molecular function level. **(B)** Top 25 of GO terms in the biological process (BP). **(C)** Top 25 of GO terms in the component (CC). **(D)** Top 25 of GO terms in the molecular function (MF).

1,373 gene information was extracted from literature records, comprehensively reflecting the genes involved in the field of neural tube malformation research. After functional enrichment analysis of these genes, it was found that at the biological process level, most genes were enriched in cellular developmental system/anatomical structure development, single-organism/organ developmental and cellular developmental; on the cellular component level, most genes are enriched in chromatin/chromosomal part, cell projection, extracellular region part and cell surface/membrane region; at the molecular functional level, most genes are related to (identical) protein binding, enzyme binding, receptor/transcription factor binding, transcriptional activator activity, sequence-specific binding, heterocyclic/organic cyclic compound binding etc. As shown in Figure 7.

**Figure 8:**
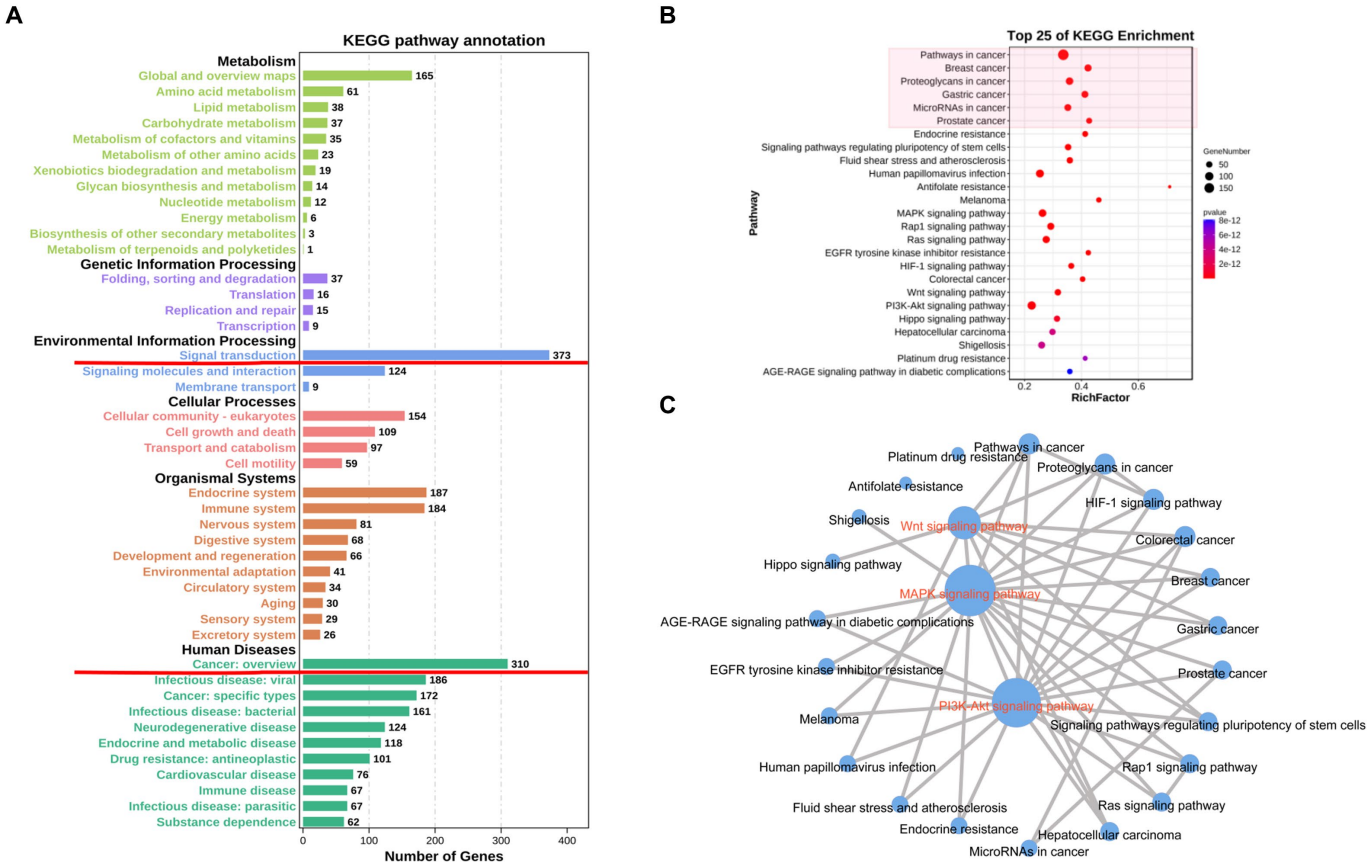
KEGG enrichment analysis result. **(A)** Statistical charts of Grade B categories of each pathway. **(B)** Top 25 of KEGG enrichment pathways. **(A,B)** shown that NTDs-related genes were mainly enriched in pathways in cancer and signal transduction. **(C)** KEGG pathways network. The signals at the center of the top 25 signaling pathway interaction network include wnt, MAPK and PI3K-Akt signaling pathways.

KEGG metabolic pathway analysis found that 1,373 genes were enriched in various cancers and signaling pathways, and the top 25 signaling pathway formed a network centered around the Wnt, MAPK and PI3K-Akt signaling pathways. As shown in Figure 8.

**Figure 9:**
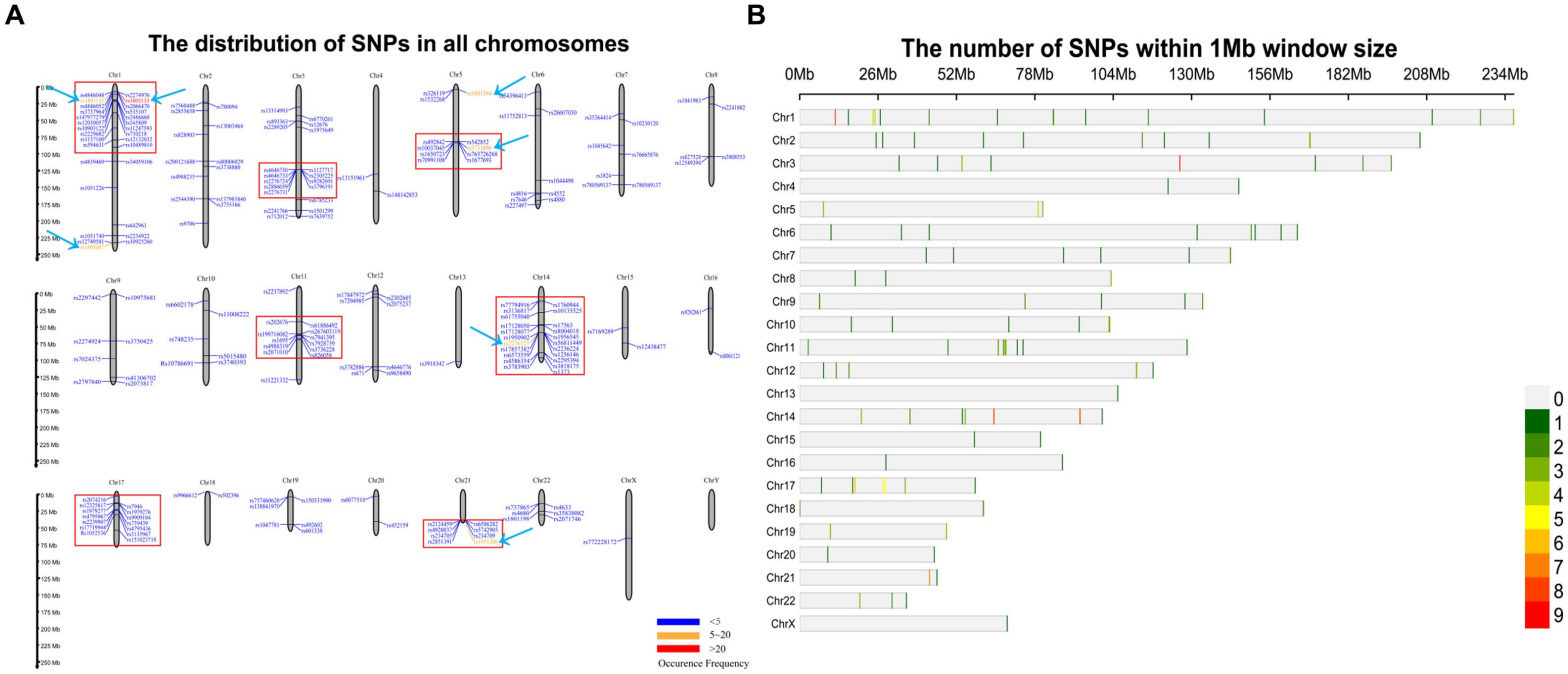
SNP distribution and density map. **(A)** SNPs distribution map. It shown that there were 7 apparent SNPs clusters framed by red lines in chromosome 1, 3, 5, 11, 14, 17, and 21. SNPs indicated by arrows with frequency greater than 5 were colored by red or orange. **(B)** SNP density plot chromosome wise representing number of SNPs within 1 Mb window size. The horizontal axis shows the chromosome length (Mb); the different color depicts SNP density.

A total of 201 SNP information were extracted from the literature records, and chromosomal localization analysis and density display were performed on these SNPs. The results indicate that there are relatively concentrated SNP clusters distributed on chromosomes 1, 3, 5, 11, 14, 17, and 21 as shown in Figure 9. 7 SNPs’ frequency greater than 5, namely rs1801133, rs2236225, rs1801131, rs1051266, rs1805087, rs1801394, and rs3733890. These SNPs that have been extensively studied are all related to folate metabolism and have been confirmed to be associated with the risk of NTDs in different populations. MTHFR rs1801133 (677 C > T), MTRR rs1801394 (66 A > G), and BHMT rs3733890 (716 G > A) maybe potential risk factors for NTD in Chinese population (Liu et al., 2014; Fang et al., 2018). MTHFR rs1801133 (677 C > T) and MTR rs1805087 (2,756 A > G) had increased risk associated with NTDs in Eastern Indian population (Kumari et al., 2022). SLC19A1 rs1051266 (80G > A) has been shown to be associated with MM risk in sample populations from Italy, the United States, and China (Findley et al., 2017). The risk of NTDs was potentially influenced by a gene–environment interaction between maternal SLC19A1 rs1051266 (80 G > A) genotype and first trimester fever. Maternal GG/GA genotype may strengthen the effect of maternal fever exposure on NTD risk in this Chinese population (Pei et al., 2015). MTHFD1 rs2236225 (1958 G > A) might be associated with maternal risk for NTDs in Caucasian populations (Jiang et al., 2014). For mothers in the lowest folate-intake group, risk of NTDs in offspring was significantly decreased for maternal MTHFR rs1801131 (1,298 A > C) (Etheredge et al., 2012). MTHFD1 rs2236225 (1958 G > A) and MTHFR rs1801131 (1,298 A > C) are in relation to anterior encephalocele susceptibility in Northeast India (Dutta et al., 2017).

## 4 Discussion

Our results provided a comprehensive overview of NTDs research throughout the world for in the last decade. NTDs research is related to the health of the birth population, involving many fields such as molecular biology, diagnostics and embryology.

In recent years, with the popularization of health care knowledge during pregnancy and the strengthening of folic acid supplementation, the incidence of NTDs has decreased significantly. However, 30–50% of NTDs are not folate preventable (Wallingford et al., 2013). And the detailed mechanism of folate prevention of NTDs still needs to be analyzed for the coming years.

Even if children with NTDs can survive after surgical correction, postoperative complications including hydrocephalus, lower limb dysfunction, urinary and fecal incontinence, sexual dysfunction seriously affect the quality of life and bring huge burden to their families and society. Therefore, the pathogenesis and preventive interventions of NTDs are still be concerned by scientists all over the world. Among mechanism studies, in addition to folate metabolism pathway, genes involved in NTDs take part in a variety of different cellular functions and biological processes, such as Wnt signaling, the planar cell polarity pathway, sonic hedgehog signaling, smad signaling, inositol pathway and so on (Harris and Juriloff, 2010; An et al., 2015; Chen et al., 2018; Liu et al., 2018; Zhang et al., 2019; Liu et al., 2020; Zohn, 2020).

The cluster analysis and trend analysis of key words show the research hotspot and direction in this field. It can be seen that there are a lot of studies on surgical treatment and management. At the same time, at the genetic level, the research on genes, proteins and mutations has always been hot, and the research on folic acid has not decreased.

We further used bioinformatics methods to analyzed the genes’ function involved in NTDs research field, and initially revealed the characteristics of gene enrichment result in this research field. Because of its relatively uniform distribution on chromosomes and much higher density than microsatellite DNA loci, SNP is considered as a new generation of genetic markers with the most application potential. It is believed that it will play an increasingly important role in the study of human complex trait diseases and pharmacogenetics in the post-genomic era (Shaw et al., 2009). In future studies, the establishment and accumulation of data on the relationship between human disease susceptibility genes and disease phenotypes and SNPs or SNPs will help us to discover the susceptibility genes that determine the genetic diseases of human complex traits. We carried out chromosome localization and distribution density analysis of the SNPs involved, which further provided the direction of further research for scientists.

Collaboration between institutions and researchers from different countries has increased significantly, which indicated that more scientific problems need the joint efforts to combat. In our study, authors and institutions with close collaboration were obtained, which can provide basic data for more extensive cooperation in the future. The multi-national collaboration network analysis revealed that authors from USA, China and UK had played an important intermediary role in the collaborative activities.

Even so, in the field of NTDs research, close cooperation between developed and developing countries is still needed, for example, to establish international joint specimen bank, to solve the problem that genetic research results based on animal models cannot be verified in human NTDs specimens. To carry out interdisciplinary research, we should not only screen gene mutations at the DNA level, but also use high-throughput omics techniques such as epigenomics, transcriptomics, proteomics and metabolomics to study molecular expression and regulation abnormalities at different levels. Identify the genes that lead to NTDs, and establish the regulatory network of these pathogenic molecules. Some clinical research findings should also be validated in larger populations, such as a study in Malaysia population, patients with spina bifida below the age of 5 were seen to have improved bladder and bowel function after the untethering surgery (Nisheljeet et al., 2022). In short, we should closely follow the frontiers, use advanced biological methods, and strengthen the interdisciplinary integration and the cooperation of different clinical research centers. This will promote the research in NTDs field, and also have important significance for its prevention and intervention of NTDs.

Bibliometrics, as a statistical research method based on literature information, has its unique advantages. It can analyze a large number of literatures at one time to find out the regularity characteristics of the given research field, which is especially important in the era of big data. As far as we know, there has never been a similar attempt in NTDs research. Our study fills the gap, gives us a macro understanding of the research trends in different countries, and the research priorities in different institutions. The information will help us to choose the most appropriate partner. However, we only analyzed the literature in the last 10 years, so it is less comprehensive and systematic. For further study, more previous literatures will be collected to construct the complete development process of NTDs research.

## 5 Conclusion

In conclusion, our findings have established that despite the huge health and social burden associated with NTDs, the international research output have not maintained a state of continuous growth. More flexible system and mechanism, open-minded international cooperation environment and perfect cross-border collaboration network is the needed of time which will help to carry out a series of scientific research. Multi-method cross analysis can reflect the core content of a certain research field. Such as our study has combined bibliometric and bioinformatics method to provided data and information for scientists, policy makers and administrators which will help them to master the macroscopic situation and make more accurate planning.

## Data availability statement

The raw data supporting the conclusions of this article will be made available by the authors, without undue reservation.

## Author contributions

RC: Conceptualization, Data curation, Formal analysis, Funding acquisition, Investigation, Methodology, Writing – original draft, Writing – review & editing. YS: Data curation, Formal analysis, Methodology, Software, Writing – original draft. RA: Investigation, Methodology, Writing – review & editing. XX: Formal analysis, Methodology, Writing – review & editing. YL: Investigation, Methodology, Writing – review & editing. ZL: Funding acquisition, Project administration, Resources, Software, Supervision, Validation, Writing – review & editing. QY: Resources, Software, Supervision, Validation, Visualization, Writing – review & editing. JX: Project administration, Resources, Software, Supervision, Validation, Writing – review & editing.

## Footnotes

^1^ https://asia.ensembl.org/info/docs/tools/vep/online/index.html

^2^ http://mg2c.iask.in/mg2c_v2.1
